# Supplementary material for: Sensing and memorising liquids with polarity-interactive ferroelectric sound
Source: Nat Commun. 2019 Aug 8;10:3575. doi: 10.1038/s41467-019-11478-1 (PMC6687823; doi:10.1038/s41467-019-11478-1)
Supplement: Supplementary file 1 — Supplementary Information [file 41467_2019_11478_MOESM1_ESM.pdf]

SUPPLEMENTARY INFORMATION

Supplementary Figures

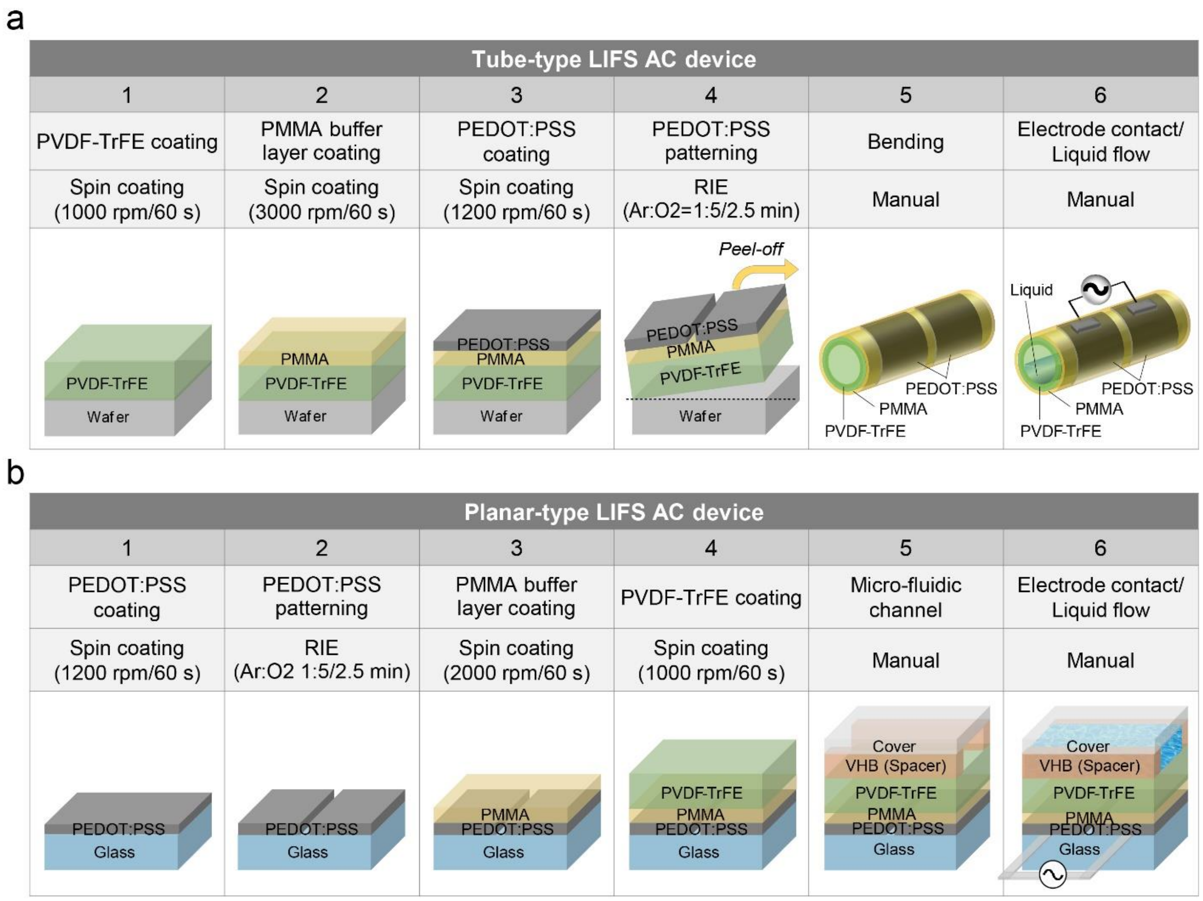

Supplementary Figure 1. Fabrication process of (a) a tube- and (b) a planar-type LIFS. In the case of the tube-type device, the detached three layers were readily deformed into a tube. In addition, the three layers were transferred to various substances such as tube, glass substrate, and human body for further applications.

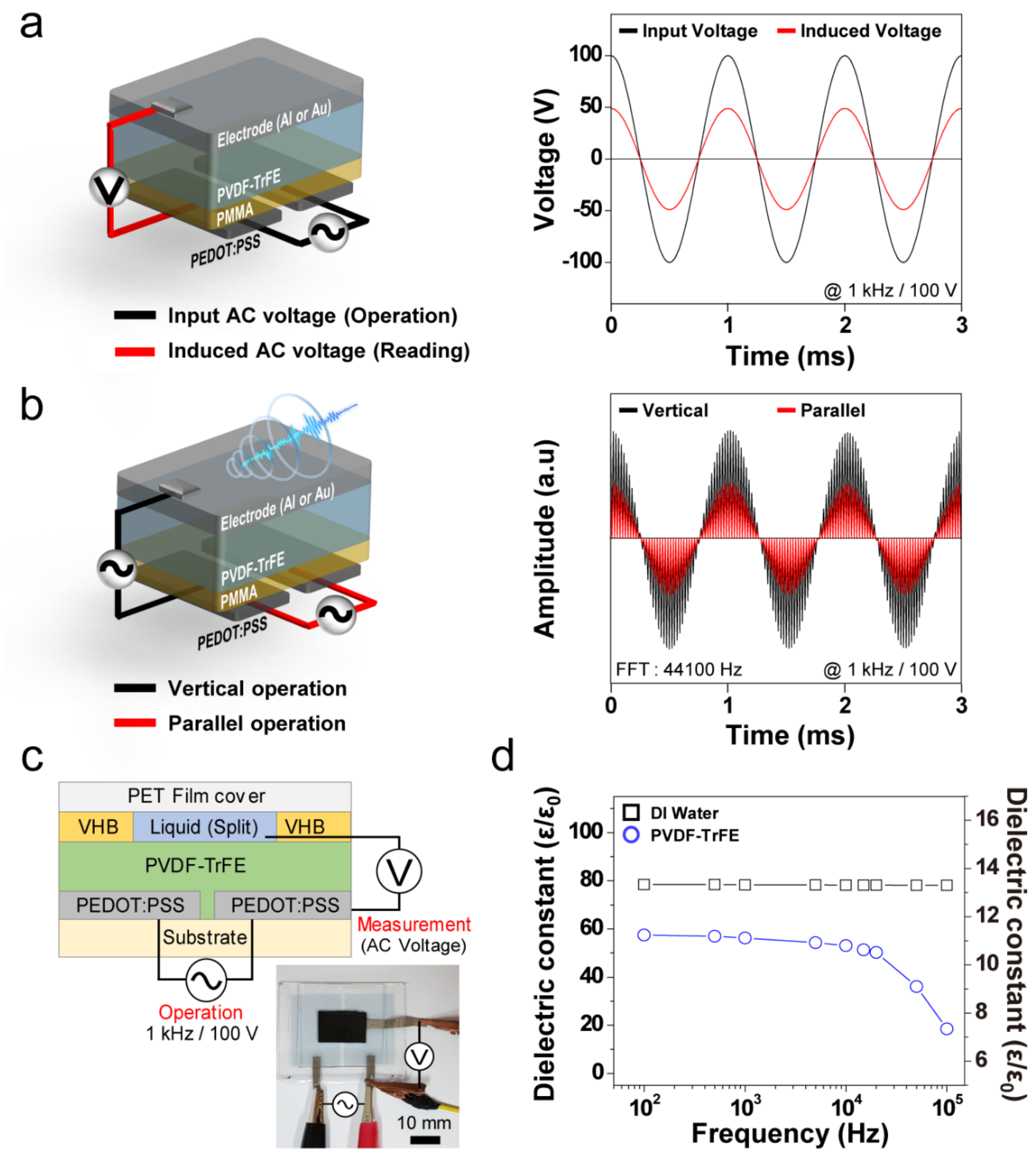

Supplementary Figure 2. Operation principle of the LIFS AC device. (a) Comparison of vertical AC voltage with in-plane applied operation voltage. AC voltage was measured between one of the bottom PEDOT:PSS electrodes and the metallic electrode deposited on the LIFS AC device. (b) Time-dependent sound amplitude with in-plane and vertical AC operation at the AC frequency of 1 kHz and the voltage of 100 V. The sound amplitudes were obtained through FFT process at 44100 Hz. (c) Schematic and photograph of the measurement of AC voltages between one of the bottom PEDOT:PSS electrodes and various liquids deposited on the LIFS AC device. (d) Dielectric constant as a function of the AC frequency of DI water and PVDF-TrFE film.

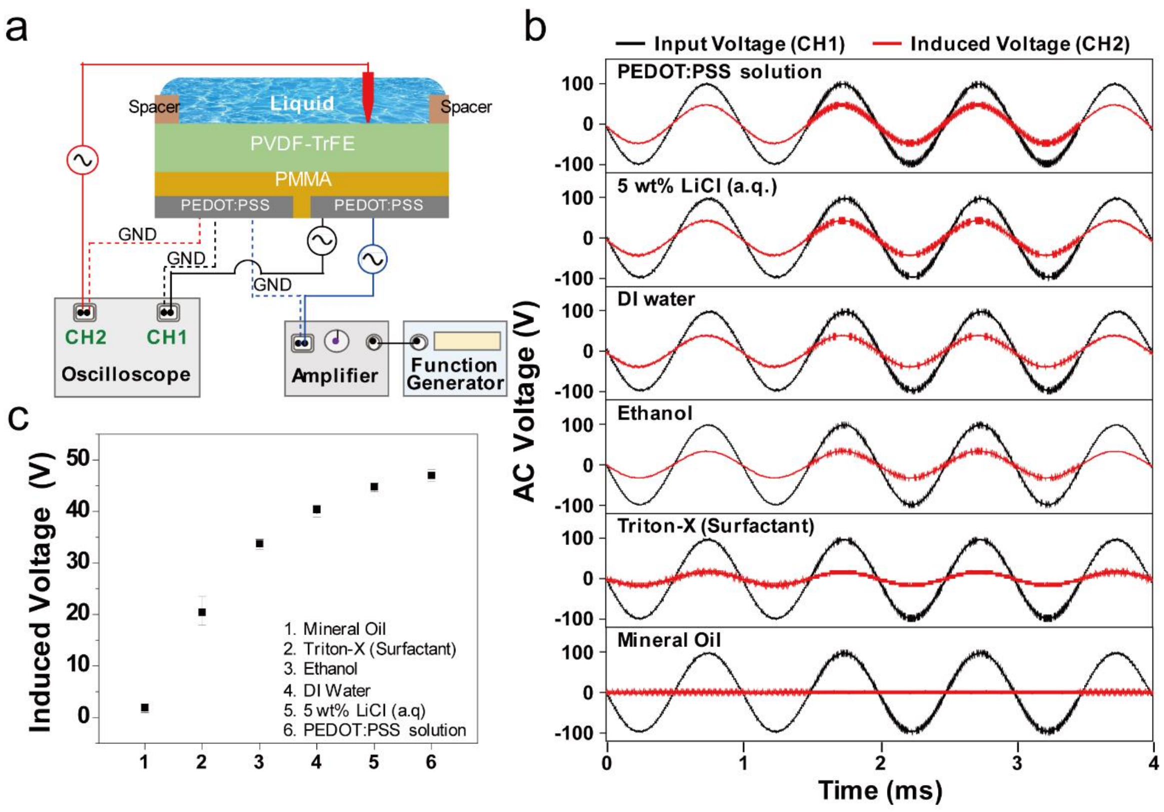

Supplementary Figure 3. Vertical AC voltage detection system. (a) Schematics of the device system between one of the bottom electrodes and various liquids deposited on the LIFS device for measuring the vertical voltage arising from a polar. (b) Time-resolved vertical voltage signals arising from various polar liquids on the LIFS AC device under an input AC voltage with a frequency of 20 kHz. (c) Vertically induced voltages arising from six liquids with different dielectric constants on an LIFS AC device. The device was operated at an AC frequency and a voltage of 20 kHz and 100 V, respectively. The values varied negligibly from one device to another, with very small error bars.

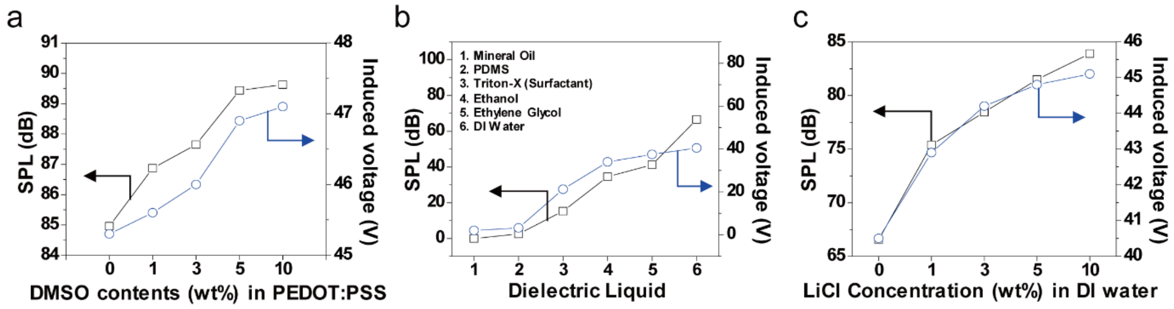

Supplementary Figure 4. Liquid-dependent SPL and vertical AC voltage of the LIFS. (a) SPL values and voltages induced by a vertical electric field arising from PEDOT:PSS with different conductivities controlled by adding DMSO on an LIFS AC device. (b) SPL values and voltages induced by a vertical electric field arising from six liquids with different dielectric constants on an LIFS AC device. (c) SPL values and voltages induced by a vertical electric field arising from different ionic conductivities controlled by the amount of LiCl in DI water on an LIFS AC device. All devices were operated at an AC frequency and voltage of 20 kHz and 100 V, respectively.

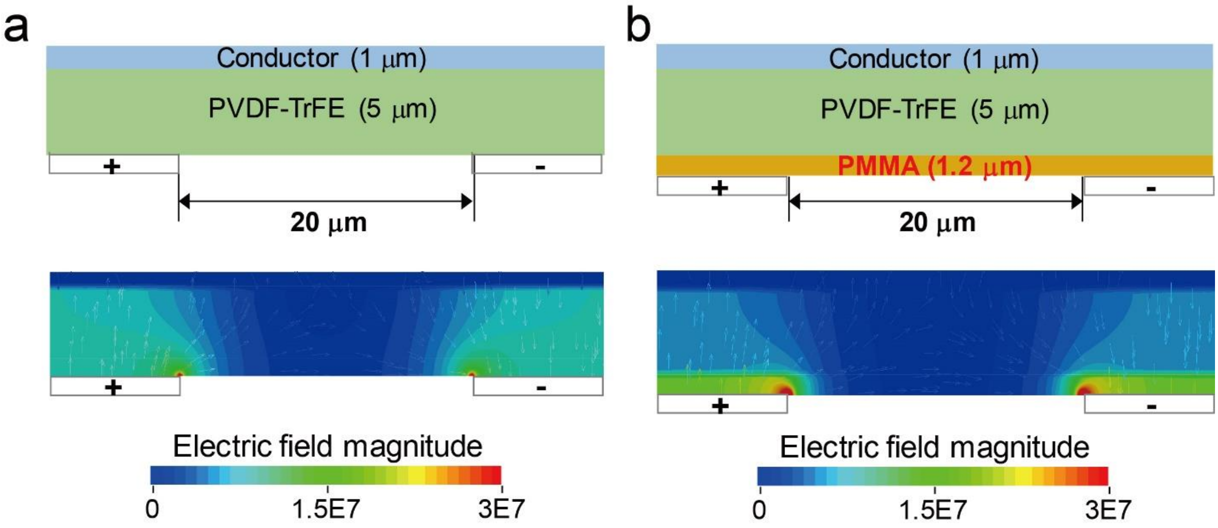

Supplementary Figure 5. Finite element method (FEM) results of the LIFS AC device. (a) PVDF-TrFE layer and (b) PMMA/PVDF-TrFE bilayer under a voltage bias between two in-plane electrodes showing both the direction and magnitude of the generated electric field with a top conductive layer on PVDF-TrFE.

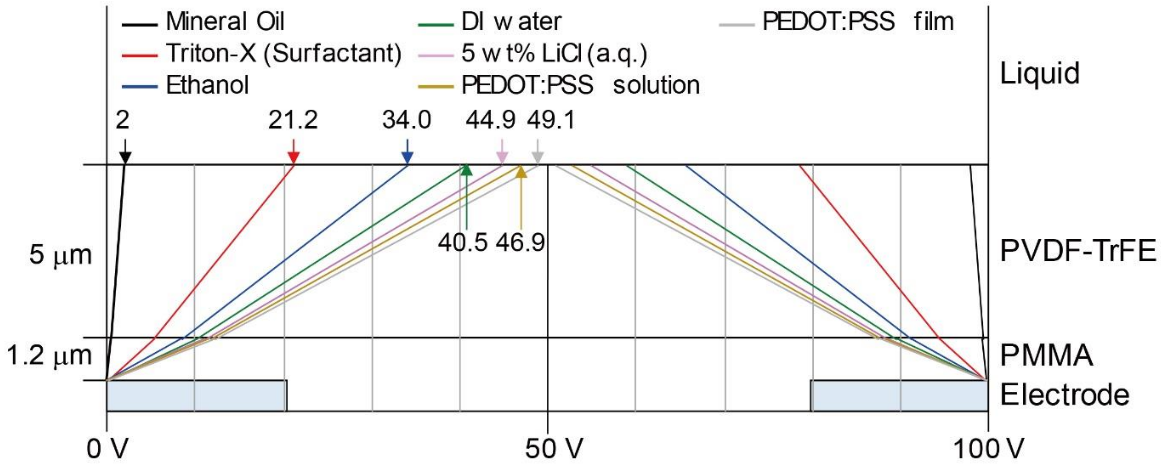

Supplementary Figure 6. Polarity-dependent vertical electric field. A simple linear electric field model on PMMA/PVDF-TrFE bilayer.

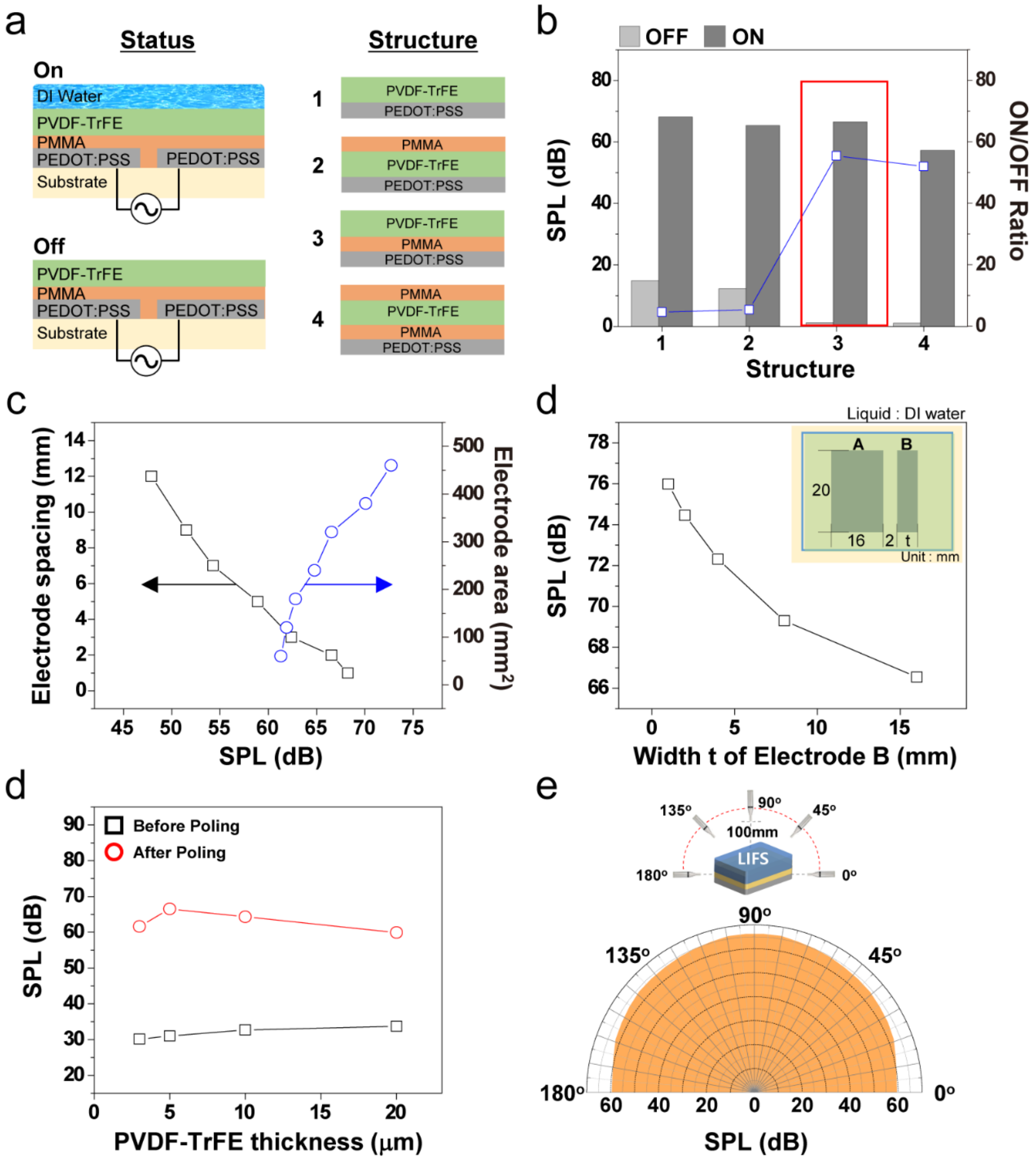

Supplementary Figure 7. Optimization of structure and sound characteristic of the LIFS. (a) Schematics of device structures before (OFF) and after (ON) liquid deposition. SPL values and ON/OFF ratios of four devices examined with different device structures schematically. (b) SPL values as functions of electrode area and spacing between the two in-plane electrodes. (c) SPL values as a function of the area ratio of the two in-plane electrodes as schematically shown in the inset. (d) SPL values as a function of the thickness of PVDF-TrFE layers before and after poling process. The poling voltage for DC poling was 2 kV. (e) Angle-dependent SPL of the LIFS AC device with DI water. The microphone is separated by 100 mm from the device. All samples are operated at the AC frequency of 20 kHz and voltage of 100 V.

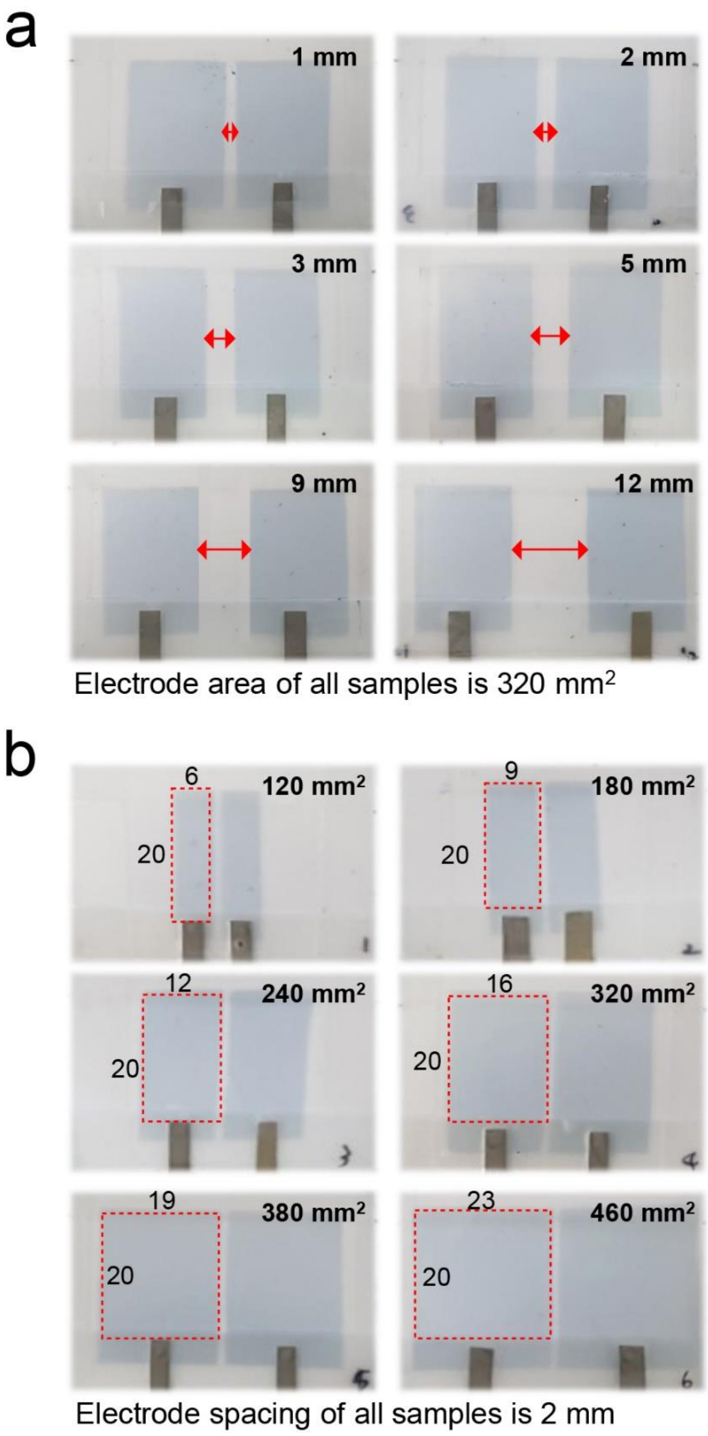

Supplementary Figure 8. Optimization of the device structure. (a) Photographs of LIFS AC devices with different dimensions for optimization of (a) electrode spacing and (b) electrode area.

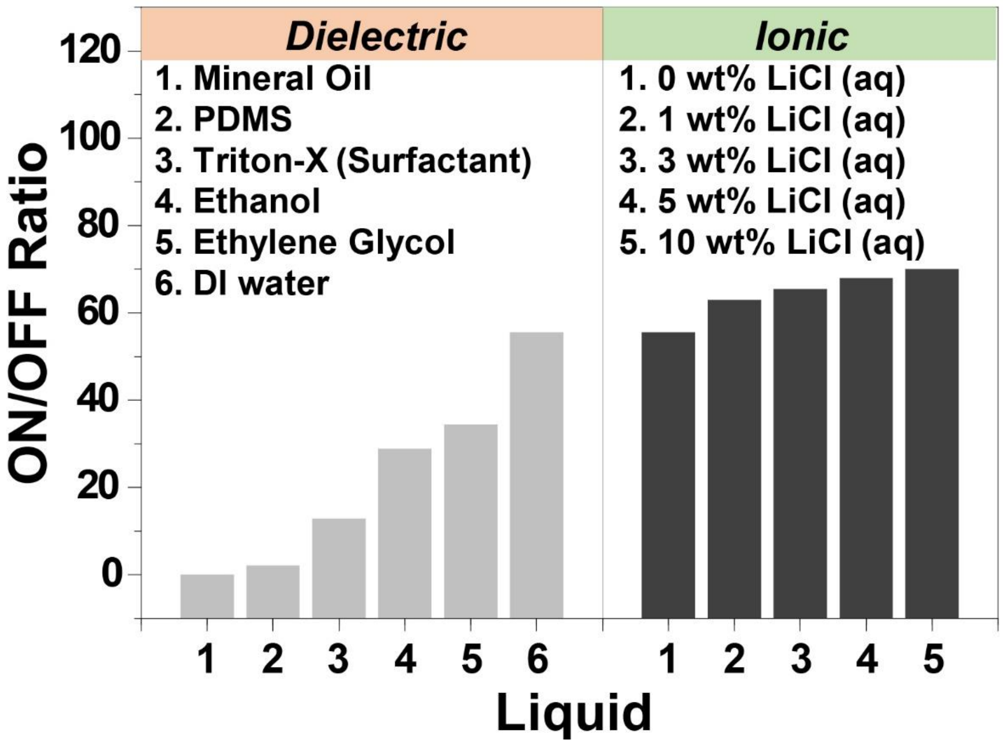

Supplementary Figure 9. ON/OFF ratios of SPL obtained from 11 liquids. The device was operated at an AC frequency and voltage of 20 kHz and 100 V, respectively.

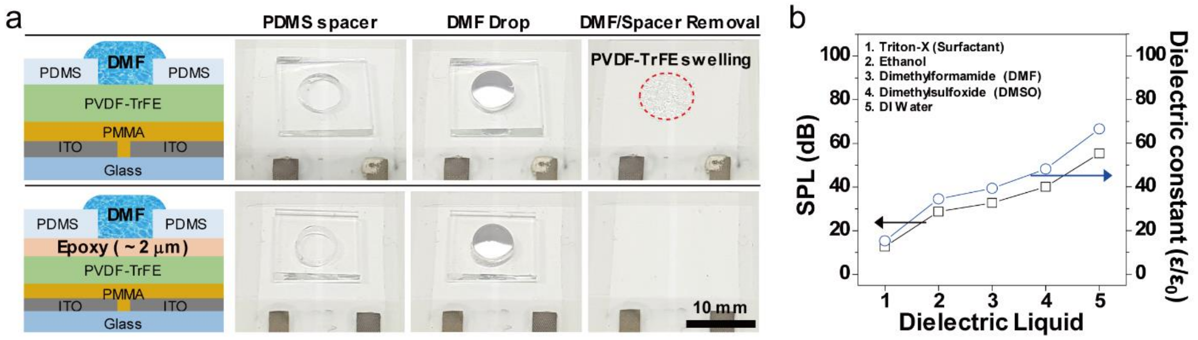

Supplementary Figure 10. LIFS with a protective layer for sensing a corrosive liquid. (a) Schematics and photographs of the device structure for the corrosive liquid sensing with and without a thin protective epoxy layer. No damage to the PVDF-TrFE layer with DMF was observed with a protective layer. (b) SPL values of five liquids including corrosive liquids such as DMF and DMSO examined with different dielectric constants on an LIFS AC device. The SPL values are proportional to the dielectric constants of the liquids. The device was operated at an AC frequency and voltage of 20 kHz and 100 V, respectively.

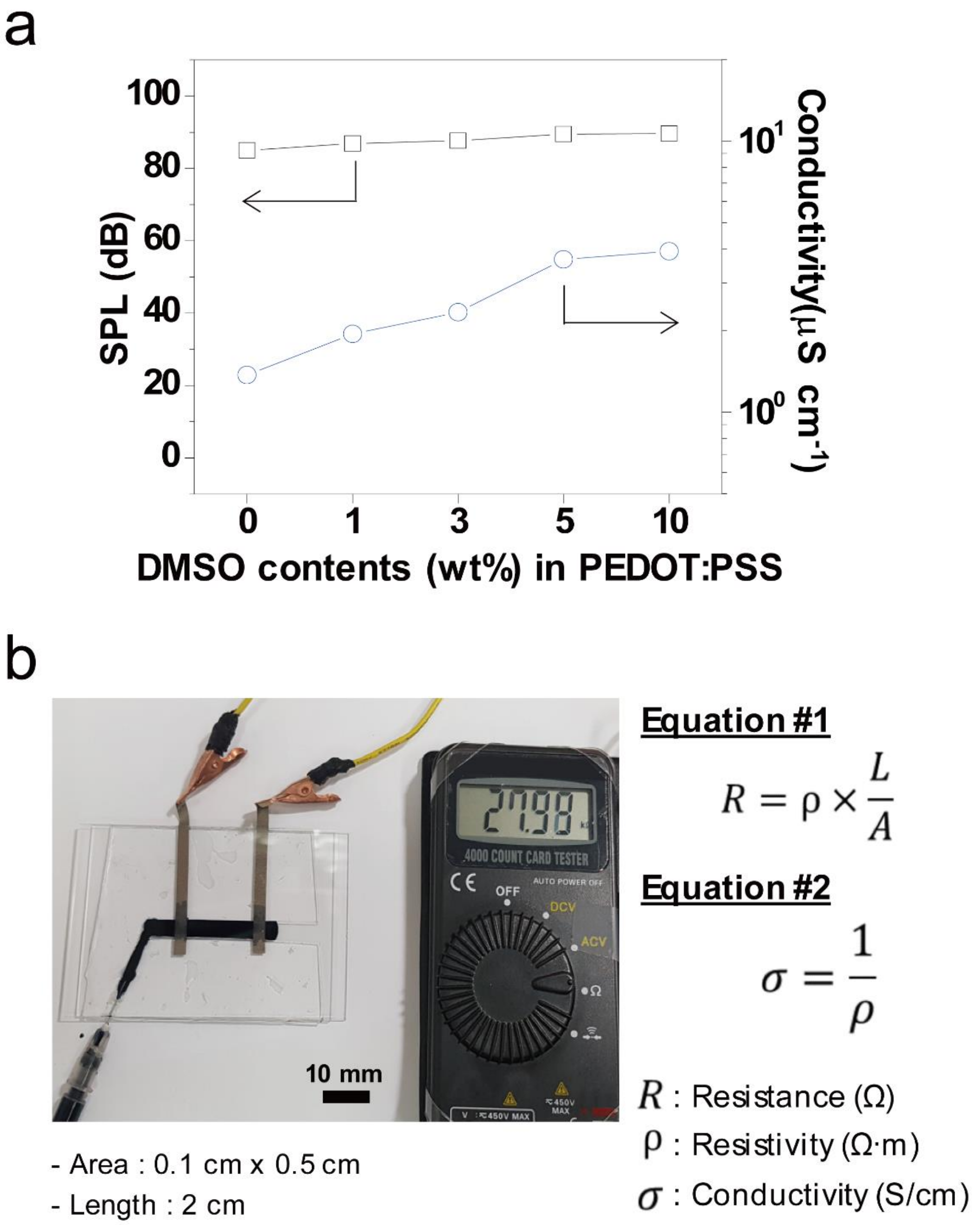

Supplementary Figure 11. Characterisation of PEDOT:PSS electrode. (a) SPL values of five liquids examined with different conductivities controlled by the amount of DMSO in PEDOT:PSS solutions on the LIFS AC device. SPL values slightly increased with the conductivities of the liquids. The device was operated at the AC frequency and voltage of 20 kHz and 100 V, respectively. (b) Photograph and equations for conductivity measurement.

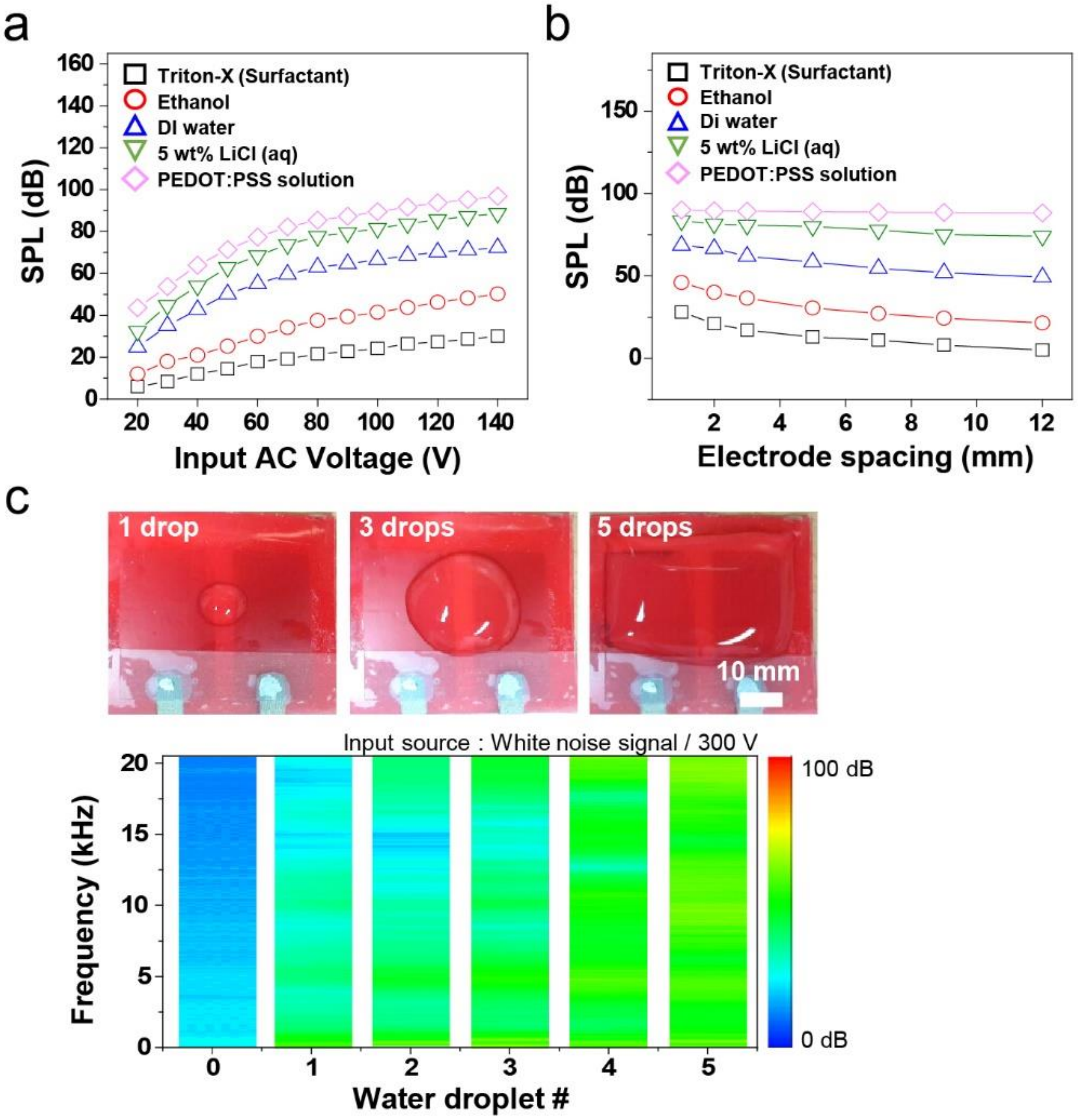

Supplementary Figure 12. Liquid-interactive sensing of the LIFS AC device. (a) SPL values of five liquids with different polarities on the LIFS AC device as a function of the voltage at the AC frequency of 20 kHz. (b) SPL values of five liquids with different polarities on the LIFS AC device as a function of the electrode spacing at the AC frequency and voltage of 20 kHz and 100 V, respectively. (c) Photographs and SPLs as a function of the number of DI water droplets. The operation condition is the AC voltage of 300 V at white noise input signal.

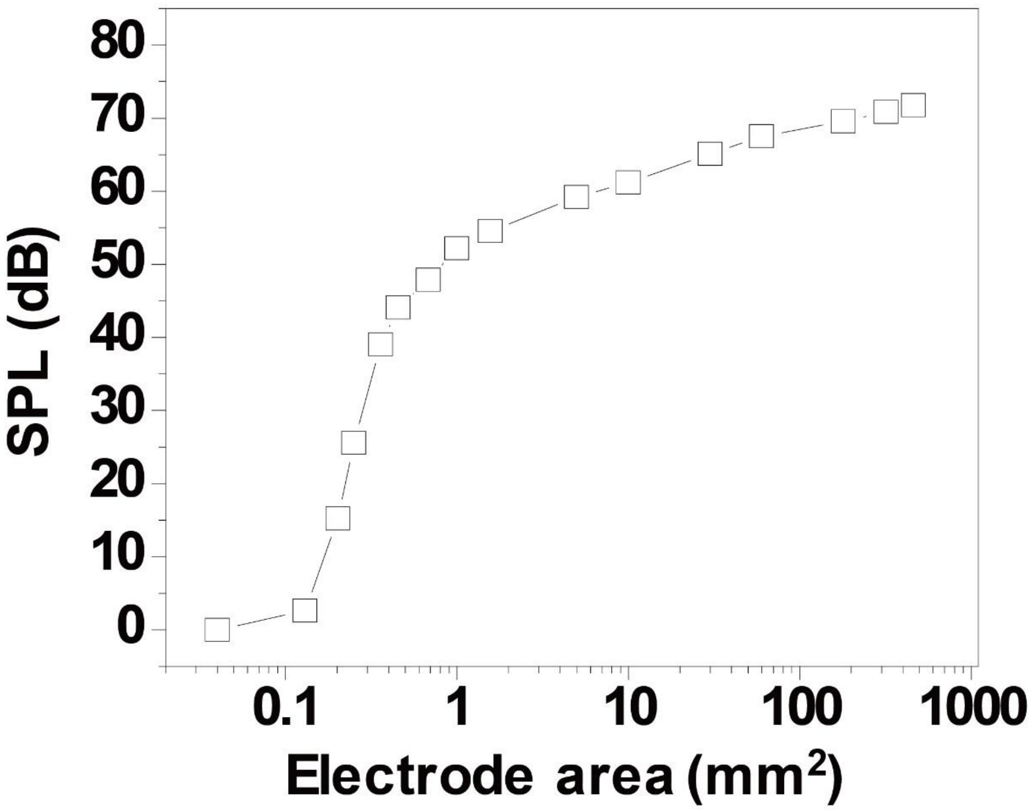

Supplementary Figure 13. SPL values with different overlapped electrode areas on an LIFS. The device was operated at an AC frequency and a voltage of 20 kHz and 100 V, respectively.

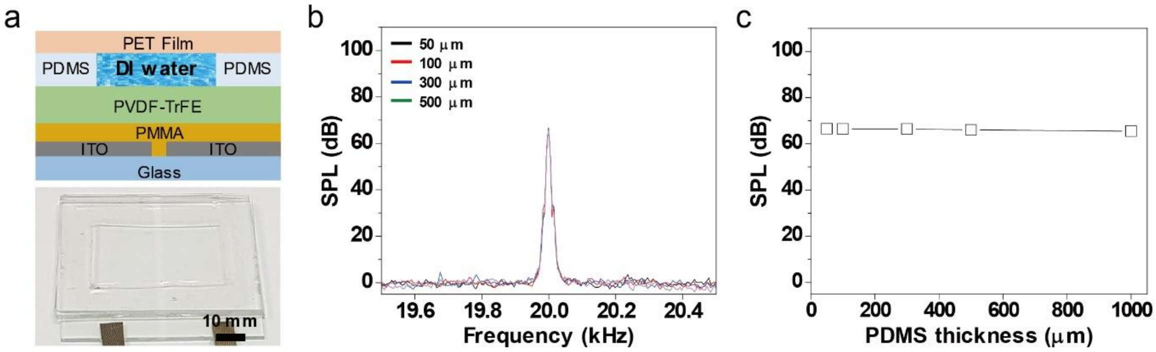

Supplementary Figure 14. SPL values of an LIFS as a function of the heights of a liquid. (a) Schematic and photograph of an LIFS AC device with DI water, whose volume can be controlled by the height of PDMS reservoir. SPL spectra (b) and values (c) arising from DI water with different heights on an LIFS AC device. The device was operated at an AC frequency and voltage of 20 kHz and 100 V, respectively.

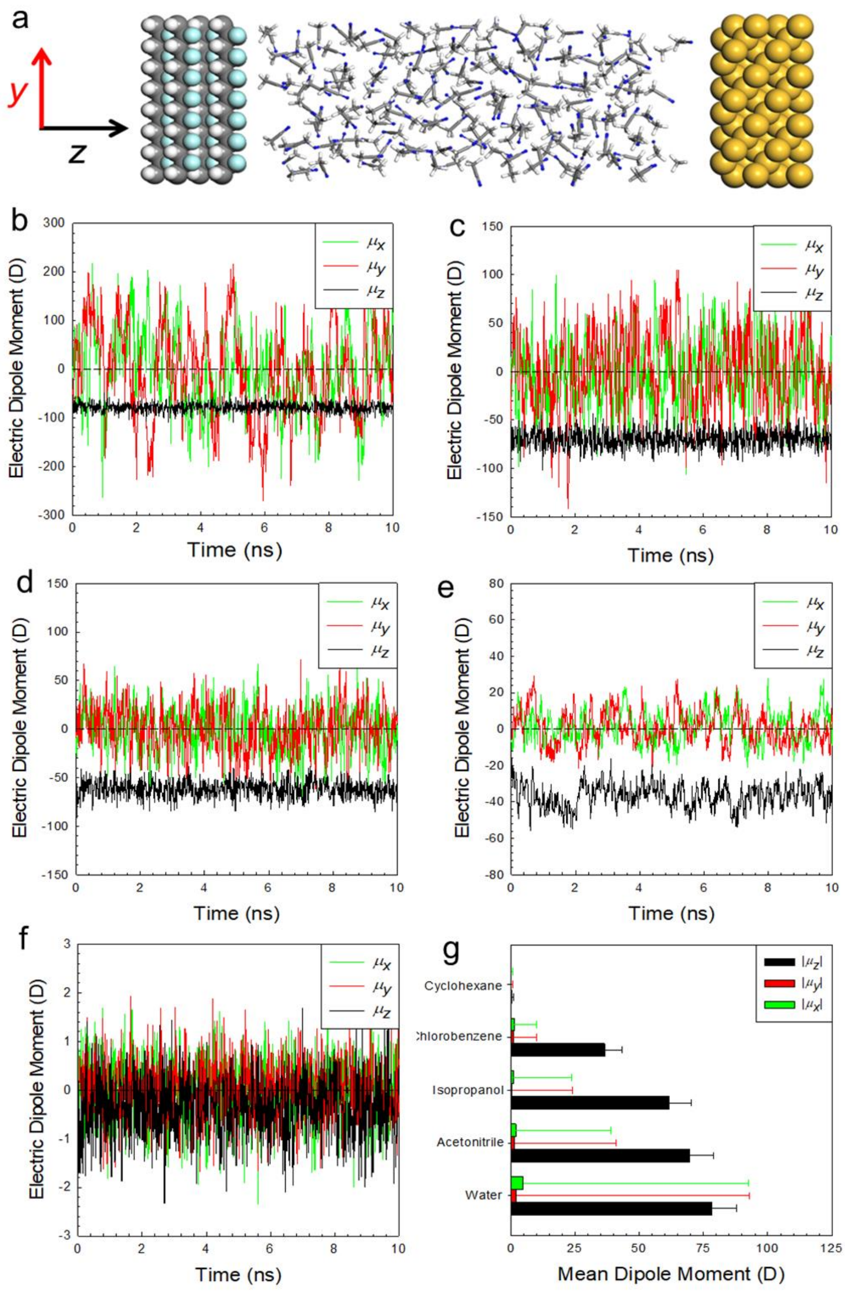

Supplementary Figure 15. Molecular dynamics simulation for liquid on PVDF. (a) The snapshot of liquid (acetonitrile) layer sandwiched between PVDF and Si layer. (b)-(f) Electric dipole moment in each direction as a function of time for (b) water, (c) acetonitrile, (d) isopropanol, (e) chlorobenzene, and (f) cyclohexane. (g) The time-average of the magnitude of dipole moment in each direction for 5 liquids (water, acetonitrile, isopropanol, chlorobenzene, cyclohexane) on PVDF.

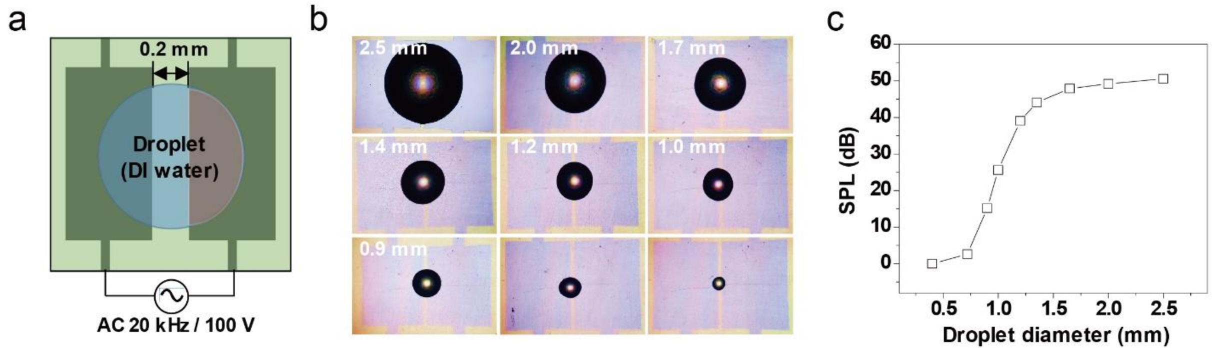

Supplementary Figure 16. Optimization of spatial resolution. (a) Schematic of a thin pad-type LIFS device with a fixed in-plane electrode gap of 0.2 mm. (b) Photographs of the LIFS device with differently sized water droplets on the electrode gap. (c) SPL values of the LIFS AC device as a function of the droplet diameter. The device was operated at an AC frequency and a voltage of 20 kHz and 100 V, respectively.

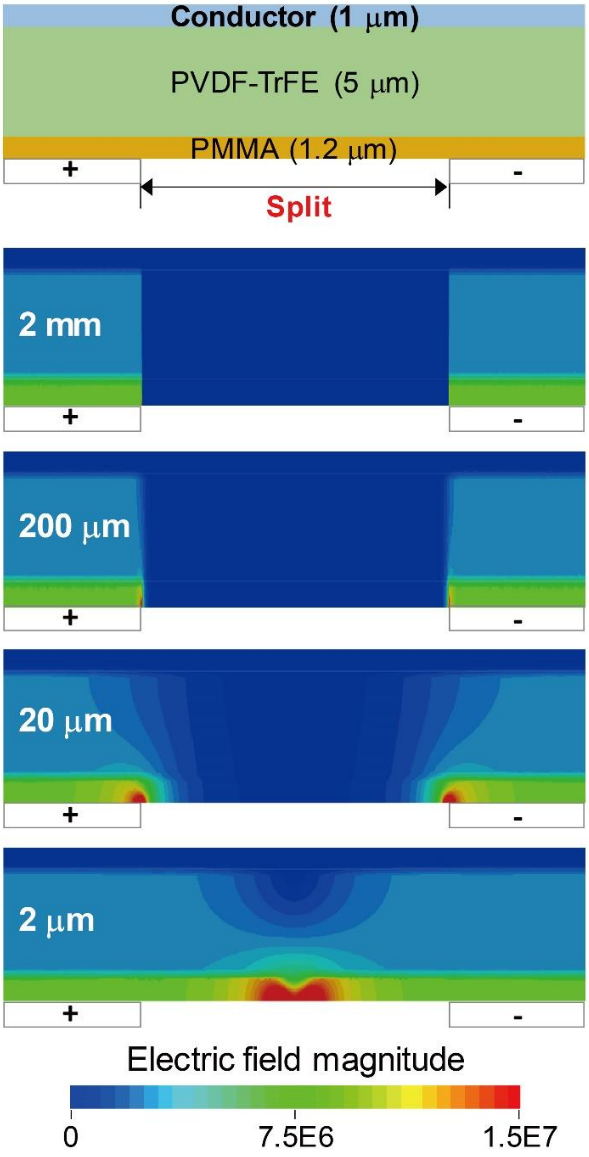

Supplementary Figure 17. Finite element method (FEM) results of the LIFS. The device under a voltage bias between two in-plane electrodes showing the magnitude of the generated electric field with a top conductive layer on PVDF-TrFE. The vertical electric field was successfully developed when the gap was 2 μm.

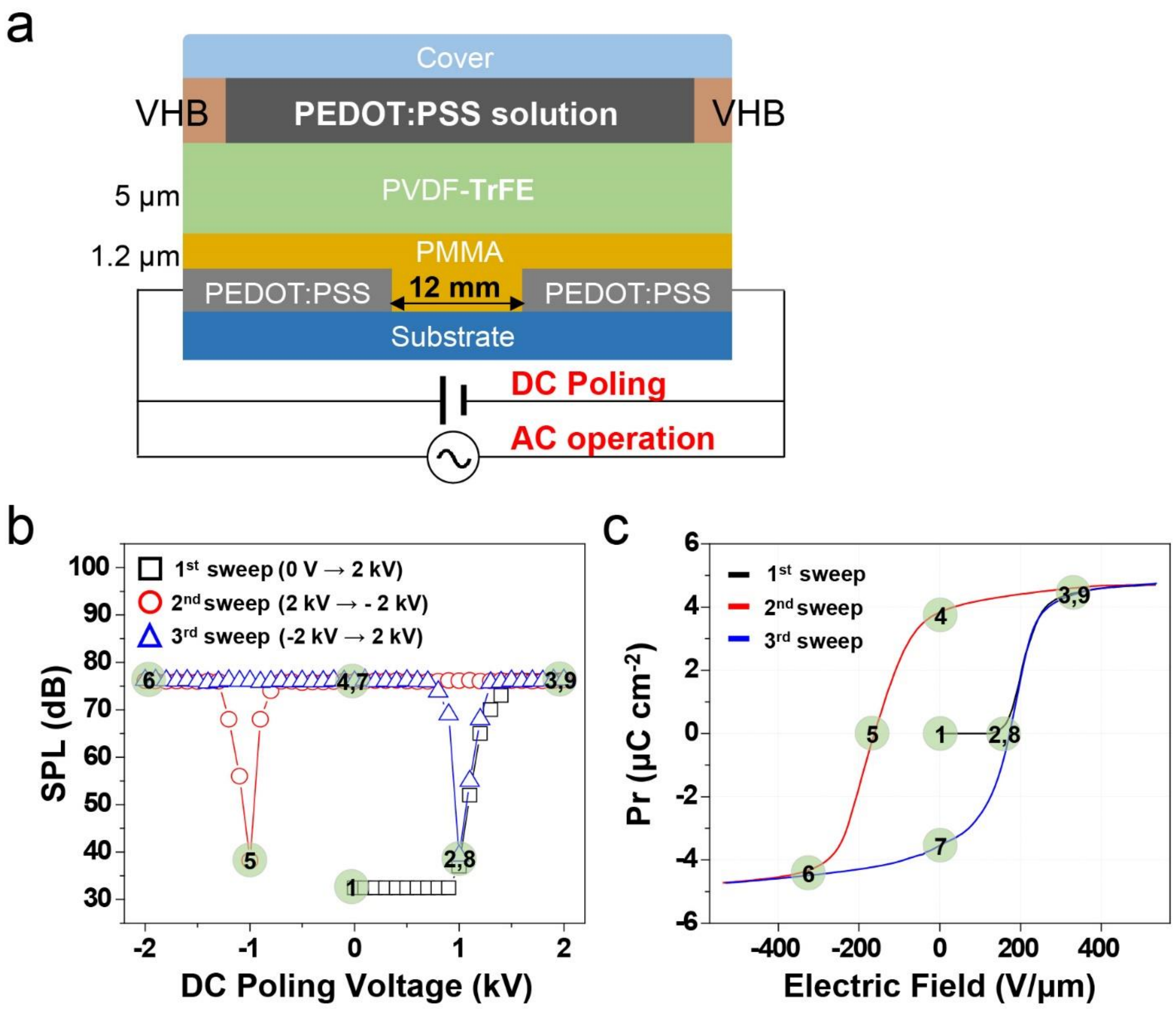

Supplementary Figure 18. Mechanism and operation circuit of non-volatile LIFS. (a) Schematics of the device structure for non-volatile LIFS AC memory. (b) A plot of SPL as a function of the DC poling voltage applied to the memory. SPL was measured at the AC frequency of 20 kHz and the voltage of 100 V. (c) The polarisation versus electric field of the LIFS AC memory. Each number (1–9) of Fig. b and Fig. c represents SPL and polarisation in the same electric field, respectively.

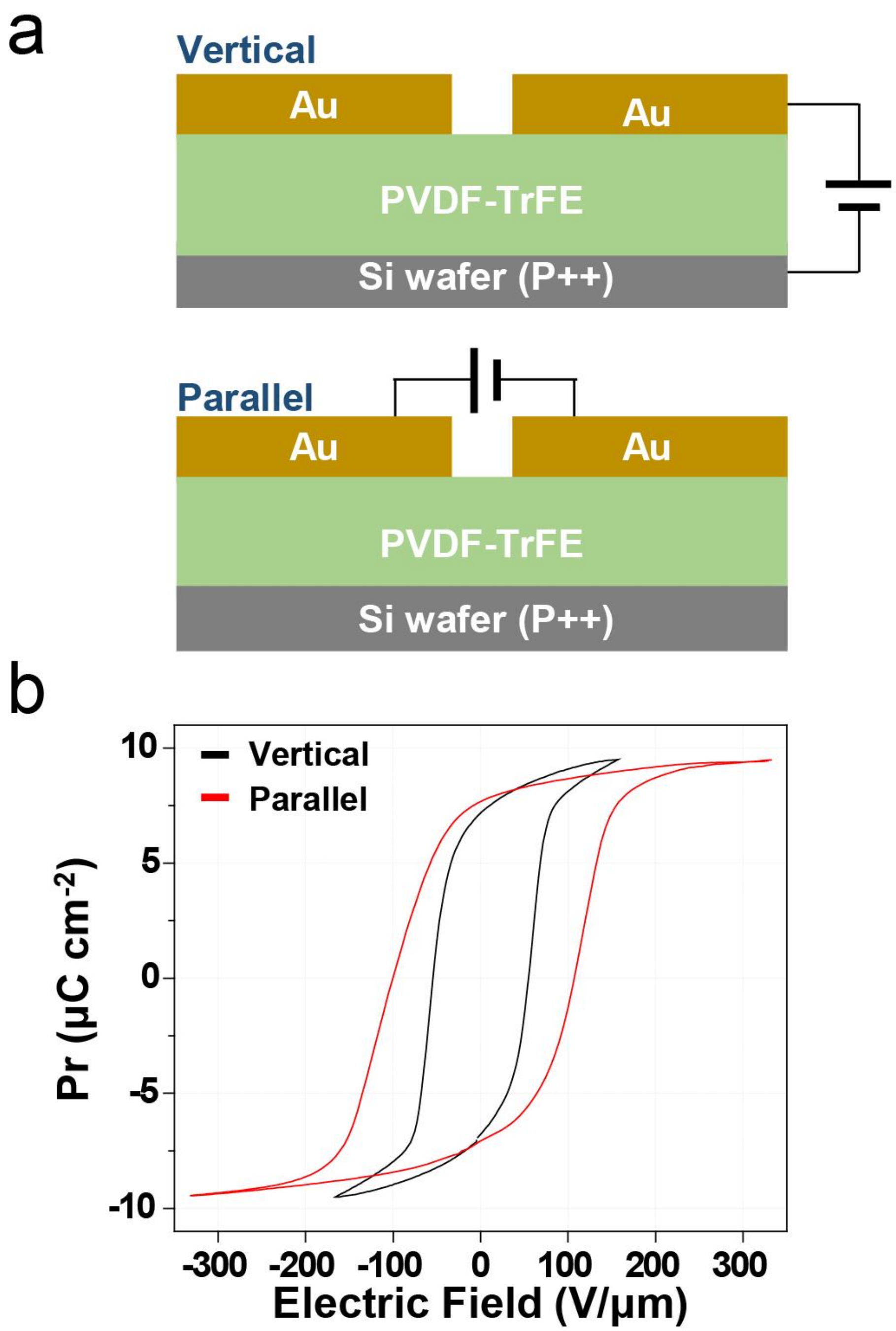

Supplementary Figure 19. Polarization behaviour of vertical and parallel type LIFS. (a) Schematics of vertical and parallel LIFS AC memory devices. (b) The polarisation versus electric field of vertical and parallel memories.

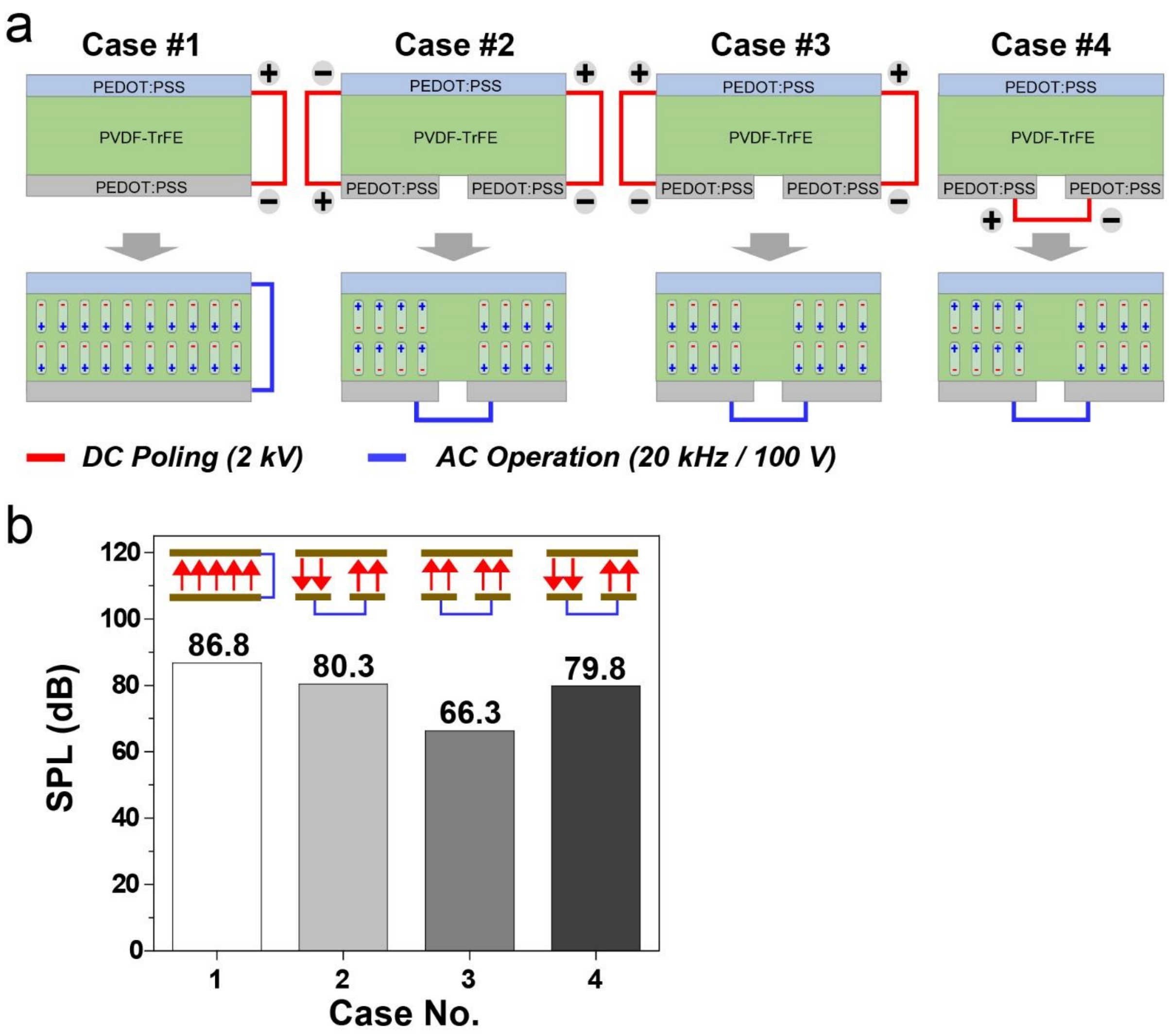

Supplementary Figure 20. Optimization of electric poling process. (a) Schematics of four different poling methods and the resulting permanent dipoles developed. (b) SPL values of LIFS AC memories with the four different poling methods upon AC operation shown in (a).

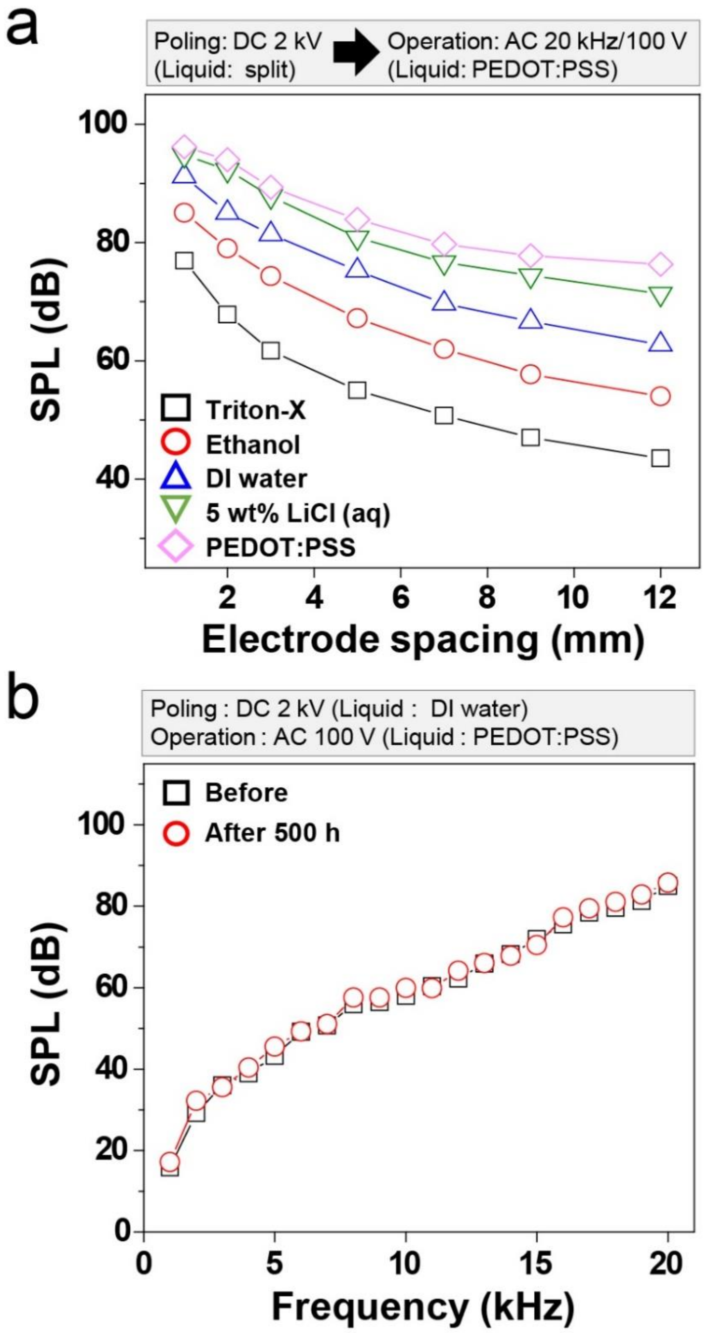

Supplementary Figure 21. SPL values and reliability over time of non-volatile LIFS. (a) SPL values of five liquids as a function of the electrode spacing. Non-volatile LIFSs were written with five liquids with different polarities and subsequently read with the reference PEDOT:PSS solutions after removal of the liquids. (b) SPL values of a non-volatile LIFS memory as a function of frequency programmed with DI and after 500 hours.

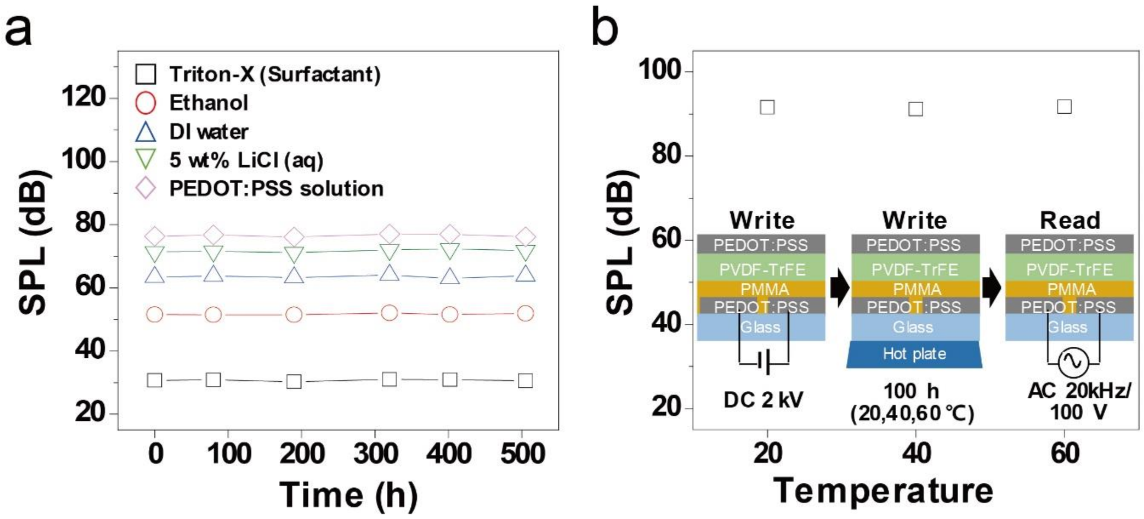

Supplementary Figure 22. Stability of non-volatile LIFS AC memory device. (a) Variation in SPL values with time arising from five liquids with different polarities on an LIFS AC device. DC voltages of 2 and −1 kV were used for writing and erasing all the liquids, respectively. The reading was obtained for all the liquids at an AC frequency and voltage of 20 kHz and 100 V, respectively. (b) Characteristic SPL value after a 100-h retention arising from deionized water at different temperatures lower than the Curie temperature of PVDF-TrFE. The reading was obtained for the water at an AC frequency and voltage of 20 kHz and 100 V, respectively.

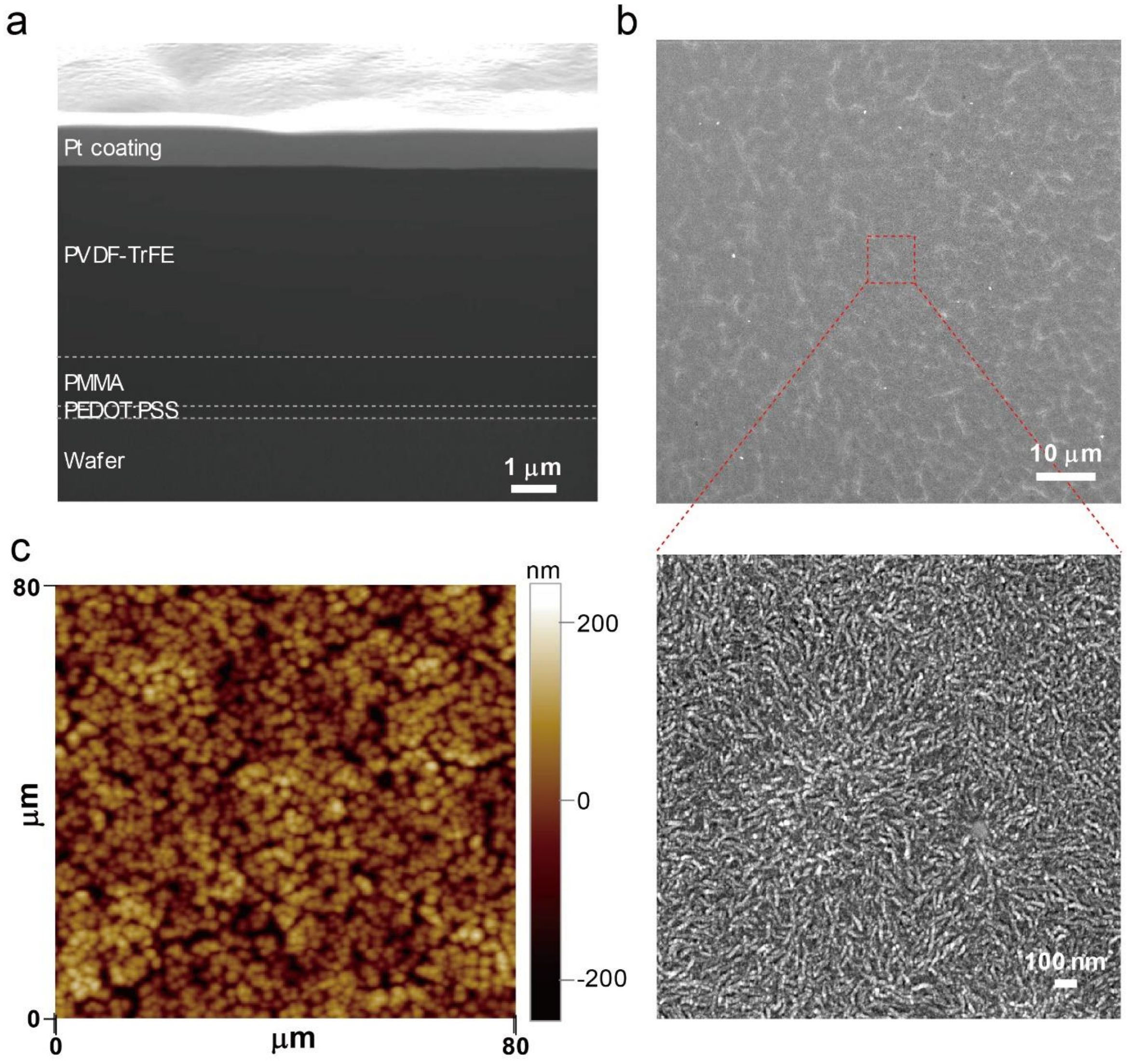

Supplementary Figure 23. Architecture and morphological analysis of the LIFS. (a) Cross-sectional scanning electron microscopy (SEM) image of an LIFS AC device. (b) SEM image of the surface morphology of the PVDF-TrFE layer. A magnitude SEM image of the regions indicated by the dotted red square shows the characteristic needle-like crystalline domains with a length of approximately 400 nm. (c) TM-AFM image of the surface morphology of the PVDF-TrFE film.

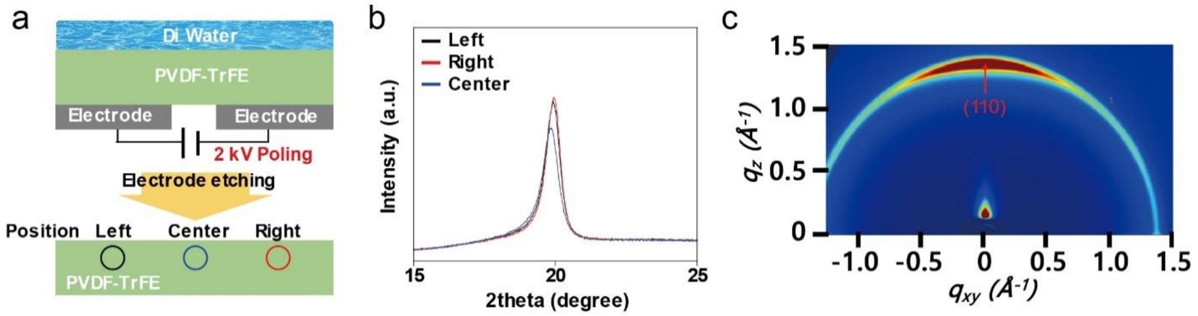

Supplementary Figure 24. Crystalline structures of a PVDF-TrFE layer in the LIFS. (a) Schematics of the cross-sectional view of the LIFS AC device with DI water on a PVDF-TrFE layer. (b) XRD patterns obtained from three different regions (left, centre, and right) of the PVDF-TrFE layer after 2 kV DC poling between the two in-plane electrodes. A higher crystallinity is clearly observed in both the left and right regions than in the centre regions. (c) 2D GIXD pattern of PVDF-TrFE layer on the LIFS AC device.

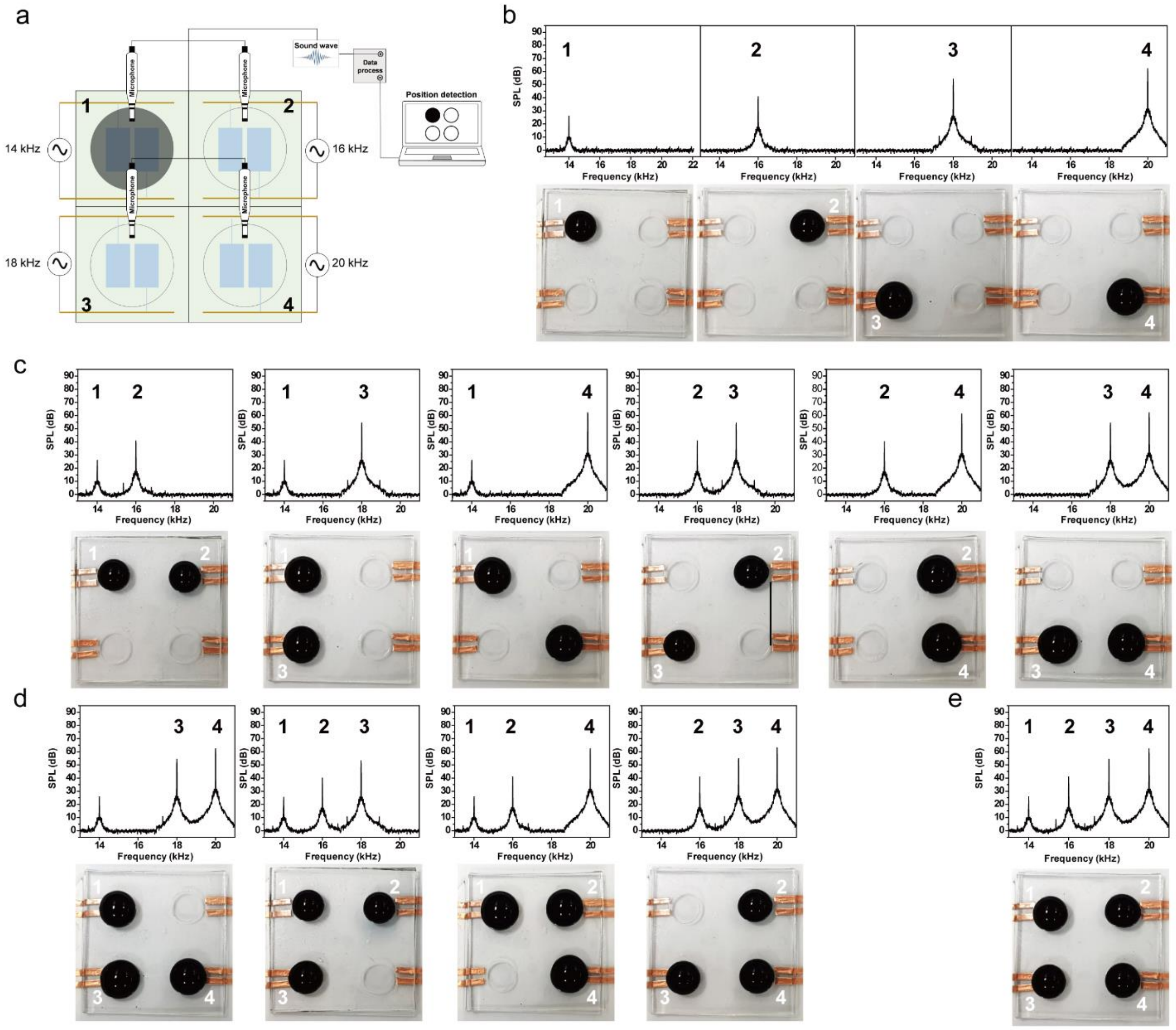

Supplementary Figure 25. Position detection of multi-droplets of a liquid on an LIFS. (a) A schematic of 2 × 2 liquid position detection pad for multi-droplet position detection. (b) SPL spectra of the four positions of each zone. Owing to the different reading frequency values of 14, 16, 18 and 20 kHz, four positions of each 2 × 2 array pad were clearly resolved in the SPL. Four different SPL spectra for a PEDOT:PSS liquid droplet were obtained, depending on the position, allowing for sound-based position detection of a liquid. (c-e) SPL spectra arising from the two, three and four PEDOT:PSS liquid droplets on the position detection pad. All SPL values were obtained at a voltage of 100 V.

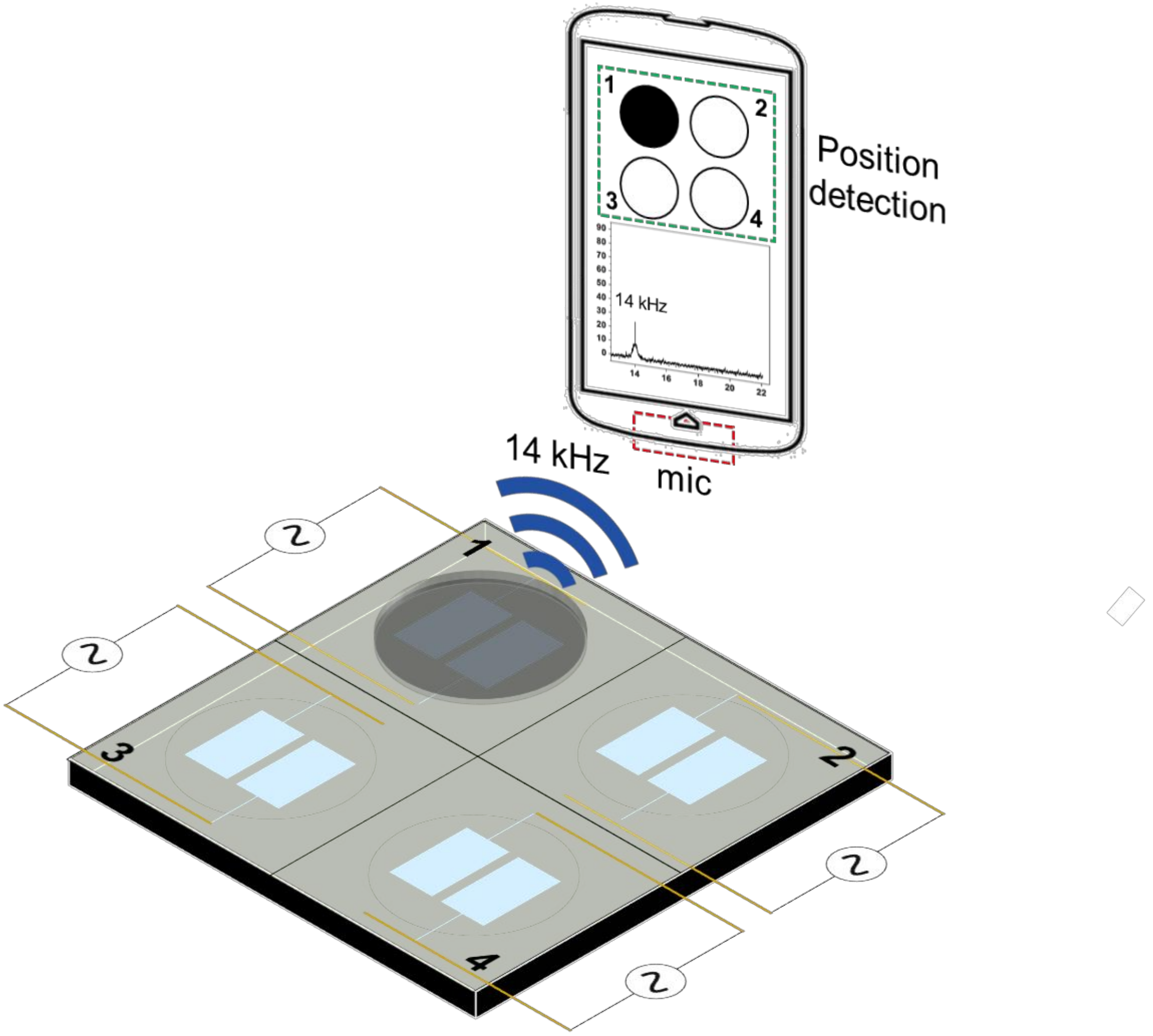

Supplementary Figure 26. A schematic of the sound detection of a liquid droplet. The system based on an LIFS with the microphone in a commercial cell-phone.

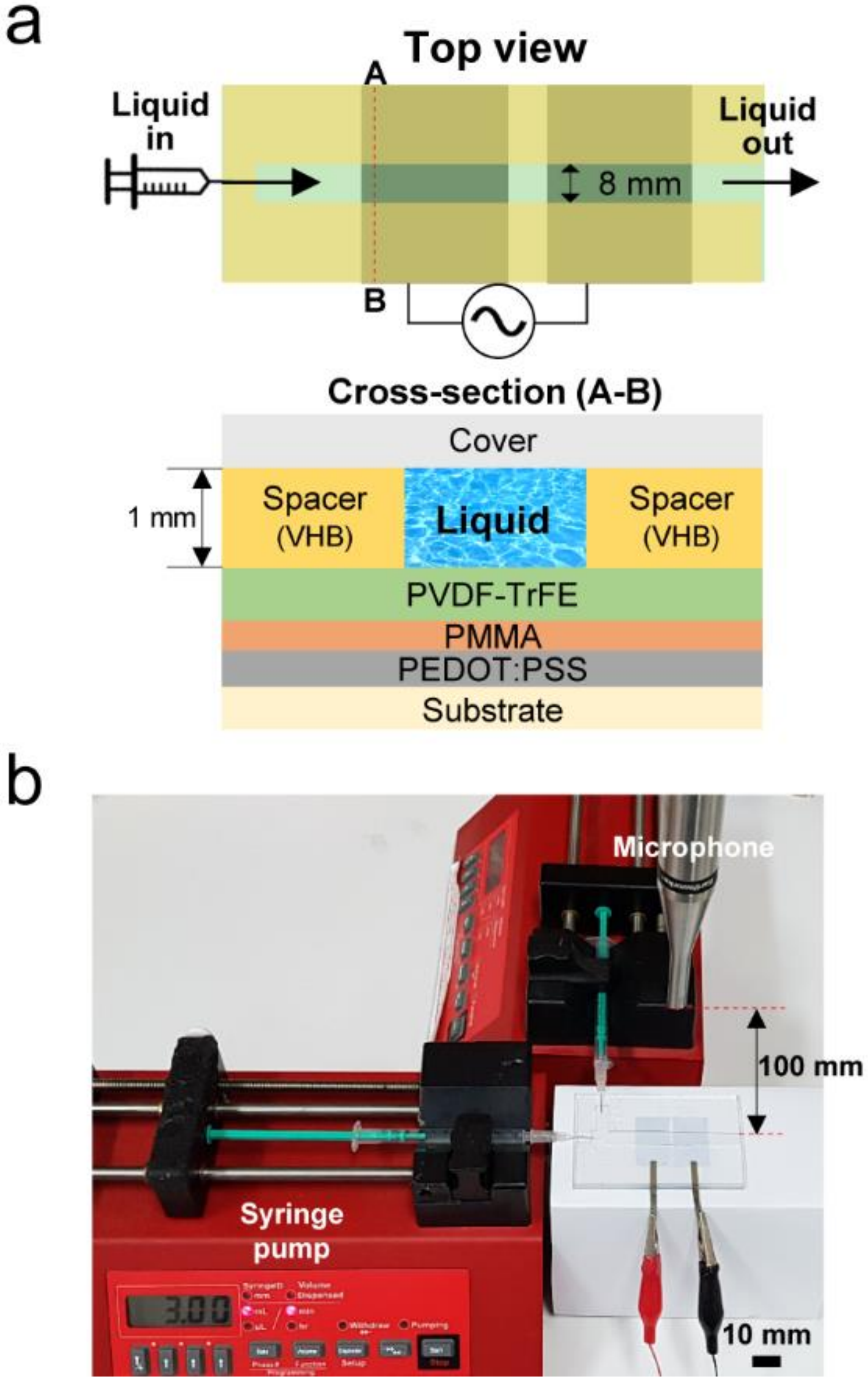

Supplementary Figure 27. Equipment set-up of an LIFS with microfluidic channel. (a) Schematics of the microfluidic channel built on an LIFS AC device. (b) A photograph of the two channel microfluidic system with an LIFS AC device for dynamic monitoring of two different liquids.

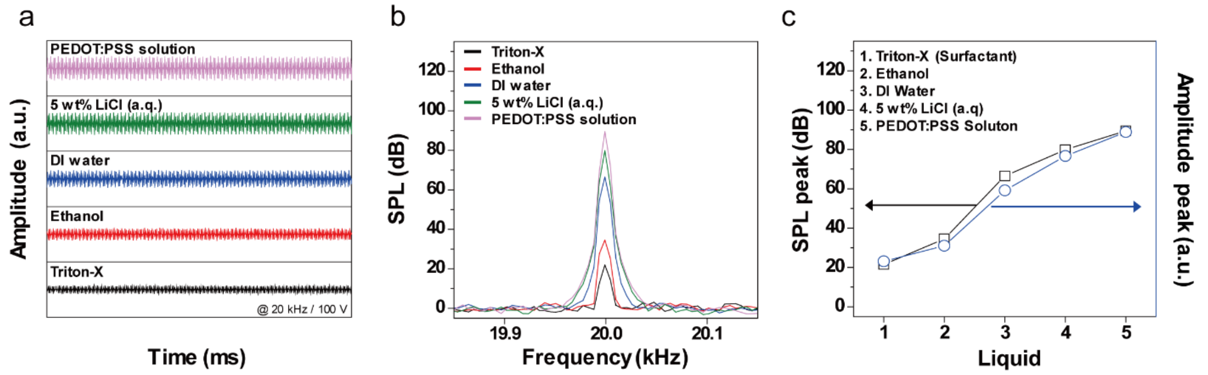

Supplementary Figure 28. Sound characterization of the LIFS AC device. (a) Amplitude of sound profile with time of five liquids having different dielectric constants at 20 KHz and 100 V. (b) SPL spectra over the whole range of audible frequency converted from the amplitude vs. time plots shown in (a) arising from the five liquids having different dielectric constants. (c) SPL values and maximum amplitude arising from the five liquids with different dielectric constants on an LIFS AC device. The device was operated at an AC frequency and voltage of 20 kHz and 100 V, respectively.

Supplementary Tables

Supplementary Table 1. Experimental conditions for fabricating the LIFS

<table>
<tr><th>Classification</th><th colspan="2">PVDF-TrFE</th><th colspan="2">PEDOT:PSS</th><th>PMMA</th></tr>
<tr><td rowspan="3">Mixing Ratio
(wt %)</td><td>PVDF-TrFE</td><td>3</td><td>PEDOT:PSS</td><td>20</td><td rowspan="4"></td></tr>
<tr><td rowspan="2">MEK
(Solvent)</td><td rowspan="2">17</td><td>DMSO</td><td>1</td></tr>
<tr><td>Surfactant</td><td>0.1</td></tr>
<tr><td>Stirring</td><td colspan="2">75 °C/450 rpm/2 h</td><td colspan="2">55 °C/550 rpm/3 h</td></tr>
<tr><td>Coating</td><td colspan="2">Spin coating
(1000 rpm / 60 s)</td><td colspan="2">Spin coating
(1200 rpm / 60 s)</td><td>Spin coating
(2000 rpm / 60 s)</td></tr>
<tr><td>Post annealing</td><td colspan="2">Hot plate (145 °C /2 h)</td><td colspan="2">Hot plate (110 °C /1 h)</td><td>Hot plate (140 °C /1 h)</td></tr>
<tr><td>Thickness</td><td colspan="2">~ 5 μm</td><td colspan="2">~ 0.4 μm</td><td>~ 1.2 μm</td></tr>
</table>

Supplementary Table 2. Characteristics of polarity-dependent sound pressure level values

No	Solvent	Polarity index	Dielectric constant (ε_r)	SPL (dB)	PVDF-TrFE solvent
1	Cyclohexane	-0.2	2.02	0	
2	Mineral oil	0.0	2.30	0	
3	n-Hexane	0.1	1.88	0	
4	Trichloroethylene	1.0	3.40	0	
5	f-Propyl ether	2.4	3.90	2.03	
6	Chlorobenzene	2.7	5.60	10.32	
7	Octoxynol-9	3.2	7.50	15.31	
8	n-Octanol	3.4	10.30	20.22	
9	n-butanol	3.9	17.50	29.23	
10	Iso-propanol	4.1	20.30	32.22	
11	Ethanol	4.3	24.60	34.52	
12	Benzonitrile	4.8	25.20	35.01	
13	Methanol	5.1	32.70	38.50	
14	Acetonitrile	5.8	37.50	40.85	
15	Dimethylformamide	6.4	36.70	-	O
16	Ethylene glycol	6.9	37.70	41.24	
17	Dimethylsulfoxide	7.2	47.00	-	O
18	DI water	10.2	78.20	66.54	

Supplementary Table 3. Vertically induced voltages arising from six liquids. The liquid-interactive ferroelectric sound device (LIFS) was operated at an alternating current (AC) frequency and a voltage of 20 kHz and 100 V, respectively.

No	Mineral Oil	Triton-X	Ethanol	DI Water	5wt% LiCl(a.q)	PEDOT:PSS
1	1	19.4	34	40.1	45	47
2	2	21.2	34.6	40.5	44.9	46.9
3	2	19.5	34	41.1	44.8	47
4	2	22.4	33.4	40.9	45.2	47.1
5	1	19.2	33.2	39.5	45.1	47
6	1.5	20.4	32.8	40.8	43.8	46.8
7	2.5	23.5	34.2	41.2	44.2	48.1
8	2.7	18.7	33.8	40.4	45.2	47.9
9	2.2	21.9	34.4	38.9	45.5	46.5
10	1.9	17.9	32.7	40.2	44.1	45.9

Supplementary Notes

Supplementary Note 1: Operation mechanism of an LIFS AC device

To understand the operation mechanism of our parallel-type LIFS AC device, first, we examined the vertical voltage when the AC voltage was applied between the two in-plane PEDOT:PSS electrodes as shown in Supplementary Figure 2a. Half of the voltage bias applied at the two PEDOT:PSS electrodes was developed between one of the bottom PEDOT:PSS and floating metal electrode due to the doubled effective thickness of the insulating layer. Subsequently, we compared the sound characteristics of the vertical and parallel device. The parallel-type device showed half of the SPL value of a conventional device with vertical contacts (Supplementary Figure 2b). Then, we investigated the AC voltage for liquids with various polarities instead of floating metal electrode (Supplementary Figure 2c). The vertical voltage built up between one of the PEDOT:PSS and liquids was significantly dependent upon the polarity of liquids and the voltage decreased with decreasing the polarity of the liquids.

Supplementary Note 2: The properties of a highly conductive PEDOT:PSS solution as a reference floating electrode

An LIFS AC device was also examined with a highly conductive PEDOT:PSS solution as a reference floating electrode. The highly conductive PEDOT:PSS solution was prepared by adding a small amount of DMSO. A series of PEDOT:PSS solutions were obtained with different conductivities by controlling the amount of DMSO in the solutions as shown in Supplementary Figure 11a. The conductivity was significantly increased with more addition of DMSO, ranging from approximately 1.37 $\mu S\ cm^{-1}$ to 3.92 $\mu S\ cm^{-1}$. The SPL was slightly enhanced with the conductivity of a floating electrode, ranging from approximately 85.0 dB to 89.6 dB. The SPL was saturated in a device with a PEDOT:PSS solution containing 5 wt% DMSO. Thus, we selected a PEDOT:PSS solution containing 5 wt% DMSO as a reference electrode. To measure the conductivity of each solution, we used the micro-fluidic system as shown in Supplementary Figure 11b. First, when the liquid flowed through the channel, the resistance between the electrode wire formed at the intervals of 2 cm was measured. Then, the resistance value was converted to the term of conductivity by the equations of Supplementary Figure 11b.

Supplementary Note 3: Molecular Dynamics Simulations

All-atom molecular dynamics (MD) simulations were performed for a model system consisting of liquid layer (water, acetonitrile, isopropanol, chlorobenzene, cyclohexane) sandwiched between PVDF and Si layer. The initial molecular geometries were prepared by placing a liquid layer with the thickness of 5 nm between PVDF and Si crystal slabs. With this system constitution, NVT-ensemble MD simulations of liquid layer were carried out at 298 K while the atomic coordinates of PVDF and Si in the upper and lower layers are fixed, using Forcite module with COMPASS force field[1] (COMPASS II) as implemented in Material Studio package. The Nose-Hoover-Langevin thermostat[2] was used for maintaining temperature (298 K). For all NVT MD runs, the electrostatic potential energy was calculated by the Ewald summation method with an accuracy of 0.1 kcal/mol and buffer width 0.5 Å (54) and the van der Waals potential energy was calculated by the atom-based technique with a cutoff distance of 12.5 Å and a spline width of 1 Å. Each of MD systems was equilibrated for 10 ns with the time step of 1 fs.

Supplementary Note 4: Mechanism of the non-volatile LIFS arising from a remnant polarization of PVDF-TrFE

To further understand the mechanism of the non-volatile memory of LIFS, we investigated the effect of remnant polarization of PVDF-TrFE on the SPL of an LIFS AC device as shown in Supplementary Figure 18. The SPL of approximately 32 dB of an un-poled LIFS (point 1 of Supplementary Figure 18b and 18c) was rapidly increased when a positive voltage was applied in the LIFS over approximately 1 kV, corresponding to approximately 160 MV/m (point 2 of Supplementary Figure 18b and 18c). As shown in Supplementary Figure 19, our parallel-type LIFS AC device required voltage two times greater than the vertical-type device. Furthermore, our LIFS AC device with PMMA layer showed a higher electric field than the device with only PVDF-TrFE alone (100 MV/m). The SPL was saturated to approximately 76 dB at the applied voltage of 1.5 kV (point 3 of Supplementary Figure 18b and 18c). The SPL was preserved even without an applied electric field due to the remnant polarization of PVDF-TrFE (point 4 of Supplementary Figure 18b and 18c). In the voltage of –1 kV, the SPL was minimized by neutralized polarization of PVDF-TrFE (point 5 of Supplementary Figure 18b and 18c). The maximum SPL was restored in the voltage of –1.5 kV as in 1.5 kV (point 6 of Supplementary Figure 18b and 18c). It implies that the SPL is not dependent on the direction of the applied electric pole such as plus or minus. However, the SPL was decreased when the polarization direction of each PVDF-TrFE on two in-plane PEDOT:PSS electrodes was deliberately formed in the same direction as shown in Supplementary Figure 20. Again, when the applied DC voltage was removed, the SPL was maintained (point 7 of Supplementary Figure 18b and 18c). In the voltage of additionally applied 1 kV, the point of 2nd SPL minimum was observed by re-neutralized polarization of PVDF-TrFE

(point 8 of Supplementary Figure 18b and 18c). when the voltage was increased, the SPL was saturated at 1.5 kV as in point 3 (point 9 of Supplementary Figure 18b and 18c). As a result, we obtained the suitable hysteresis curve for memory application.

Supplementary References

1. Sun, H. An ab Initio Force-Field Optimized for Condensed-Phase Applications - Overview with Details on Alkane and Benzene Compounds. *J. Phys. Chem. B* **102**, 7338-7364 (1998).

2. Samoletov, A.A. *et al*. Thermostats for "Slow" Configurational Modes. *J. Stat. Phys.* **28**, 1321-1336 (2007).
