# Supplementary material for: Sensing and memorising liquids with polarity-interactive ferroelectric sound
Source: Nat Commun. 2019 Aug 8;10:3575. doi: 10.1038/s41467-019-11478-1 (PMC6687823; doi:10.1038/s41467-019-11478-1)
Supplement: Supplementary file 3 — Description of Additional Supplementary Files [file 41467_2019_11478_MOESM3_ESM.pdf]

Description of Additional Supplementary Files

File Name: Supplementary Movie 1
Description: A planar-type LIFS AC device as a function of amount of liquid

File Name: Supplementary Movie 2
Description: A planar-type LIFS AC device as a function of position of a liquid droplet

File Name: Supplementary Movie 3
Description: A tube-type LIFS AC device with deionized water passing through the tube

File Name: Supplementary Movie 4
Description: A tube-type LIFS AC device with attachable electrodes containing deionized water
